# Fault Diagnosis of Rolling Bearings in Primary Mine Fans under Sample Imbalance Conditions

**DOI:** 10.3390/e25081233

**Published:** 2023-08-18

**Authors:** Wei Cui, Jun Ding, Guoying Meng, Zhengyan Lv, Yahui Feng, Aiming Wang, Xingwei Wan

**Affiliations:** 1 School of Mechanical Electronic & Information Engineering, China University of Mining & Technology (Beijing), Beijing 100083, China; bqt1900402009@student.cumtb.edu.cn (W.C.); mgy@cumtb.edu.cn (G.M.); zqt2000401017@student.cumtb.edu.cn (Z.L.); 2010490107@student.cumtb.edu.cn (Y.F.); 201418@cumtb.edu.cn (A.W.); bqt2000401005@student.cumtb.edu.cn (X.W.); 2 School of Emergency Equipment, North China Institute of Science and Technology, Langfang 065201, China

**Keywords:** primary mine fan, rolling bearing, sample imbalance, image generation, fault diagnosis

## Abstract

Rolling bearings are crucial parts of primary mine fans. In order to guarantee the safety of coal mine production, primary mine fans commonly work during regular operation and are immediately shut down for repair in case of failure. This causes the sample imbalance phenomenon in fault diagnosis (FD), i.e., there are many more normal state samples than faulty ones, seriously affecting the precision of FD. Therefore, the current study presents an FD approach for the rolling bearings of primary mine fans under sample imbalance conditions via symmetrized dot pattern (SDP) images, denoising diffusion probabilistic models (DDPMs), the image generation method, and a convolutional neural network (CNN). First, the 1D bearing vibration signal was transformed into an SDP image with significant characteristics, and the DDPM was employed to create a generated image with similar feature distributions to the real fault image of the minority class. Then, the generated images were supplemented into the imbalanced dataset for data augmentation to balance the minority class samples with the majority ones. Finally, a CNN was utilized as a fault diagnosis model to identify and detect the rolling bearings’ operating conditions. In order to assess the efficiency of the presented method, experiments were performed using the regular rolling bearing dataset and primary mine fan rolling bearing data under actual operating situations. The experimental results indicate that the presented method can more efficiently fit the real image samples’ feature distribution and generate image samples with higher similarity than other commonly used methods. Moreover, the diagnostic precision of the FD model can be effectively enhanced by gradually expanding and enhancing the unbalanced dataset.

## 1. Introduction

Primary mine fans are essential pieces of equipment in mine ventilation systems, and they are key pieces of mechanical equipment that guarantee safe coal production [1,2]. A primary mine fan provides sufficient fresh air and regulates the temperature and humidity of the underground air at each operating location. Simultaneously, it dilutes and discharges toxic and harmful underground gases and dust and has the features of high levels of power and long operation times [3,4,5]. The normal operation of a primary mine fan significantly affects the safety of personnel, production safety, production efficiency, and many other aspects of coal mine production.

**Figure 1:**
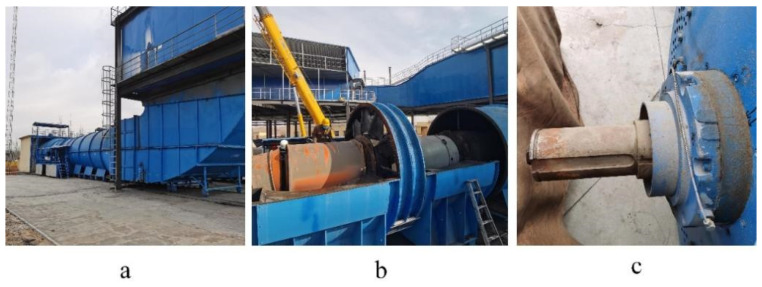
Primary coal mine fan: (**a**) axial-flow fan, main part; (**b**) explosion-proof motor; (**c**) motor connection axis extension.

A primary mine fan usually employs an axial-flow ventilation fan and a high-voltage, explosion-proof, and three-phase asynchronous motor as the matching motor. The fan is directly connected to the motor, as shown in Figure 1. The rolling bearing (RB) is a key part of the main fan [6], which is the bridge between the rotating and stationary parts of the fan. The rolling bearings are subjected to large axial and radial loads and withstand the dynamic load caused by pneumatic imbalances during the fan’s operation. Therefore, rolling bearing faults can cause damage to the main fan, and this risk cannot be ignored. The rolling bearings’ condition directly influences the equipment’s operation, which can cause extensive and prolonged production halts once a failure occurs. This affects a coal mine’s production safety and economic efficiency, which can even lead to catastrophic coal mine accidents in serious cases. Therefore, studying rolling bearings’ condition monitoring and fault diagnosis (FD) in primary mine fans is necessary.

With the increasing growth of computer technology and sensor and signal processing technologies, various studies have been performed on the FD of rolling bearings. Sun et al. presented a bearing FD approach using empirical modal decomposition (EMD) and improved Chebyshev distance [7]. Du et al. proposed a sparse time-frequency analysis approach using a first-order primal-pair algorithm to obtain sparse time-frequency images employed for extracting texture features as input features for the classifier. They classified bearing faults using a grayscale co-occurrence matrix [8]. Li et al. presented an FD approach using improved multiscale symbolic dynamic entropy (MMDE) and minimum redundancy maximum relevance (mRMR) for identifying the different health states of planetary gearboxes [9].

With the rapid development of industrial technology, the volume of monitoring data for key components, such as rolling bearings and gearboxes, has increased substantially. Because of the small parameter space, traditional shallow machine learning methods are prone to falling into local extreme points during training and cannot better process a large number of monitoring signals, and the shallow model has high requirements for the input data. The diagnostic performance of the model is highly reliant on signal processing and feature extraction techniques. This leads to difficult training and poor generalization of fault diagnosis models [10]. Deep learning methods can automatically learn features from raw inputs and process them. The focus of deep learning-based fault diagnosis research is to automatically extract representative fault features from complex monitoring signals to characterize the operating state of the diagnostic target and reduce the impact of manual feature extraction on the accuracy of fault diagnosis [11]. Deep learning-based fault diagnosis methods solve the problem of high workload and high cost when feature extraction is performed on large volumes of complex monitoring data by constructing end-to-end diagnostic models that directly establish a link between the growing amount of monitoring data and the health of the machine. It also has the advantages of excellent fault feature extraction capability, high diagnostic accuracy, and strong generalization [12]. Thus, with the breakthroughs in deep learning (DL) research, many scholars have released their reliance on signal processing techniques and manual diagnostic experience and diagnosed faults in rolling bearings by training deep learning models through increasing monitoring data. Gu et al. utilized CEEMDAN to denoise and reconstruct the angular domain vibration signals, visualized the denoised reconstructed signals using SDP, and classified the constructed SDP patterns using DCNN to perform FD [13]. Wang et al. constructed a new network framework (SVD-1DCNN) using singular value decomposition (SVD) and a one-dimensional convolutional neural network (1DCNN) to achieve the intelligent detection of bearing faults with the raw signal as input [14]. Huang et al. proposed a multiscale CNN with channel attention (CA-MCNN) that extracts the multiscale information of bearing signals using maximum and average pooling layers and utilizes a one-dimensional convolution-based feature parallel fusion mechanism for capturing complementary multiscale information and alleviating network complexity. In addition, they introduced a channel attention mechanism to enhance the convolutional layer’s feature learning capability [15]. Sun et al. established an optimized CNN model for FD, determining the convolutional neural network model based on new metrics containing an accuracy and time ratio by adjusting the number of convolutional layers and the convolutional kernel size [16]. Xue et al. established a two-stream feature fusion CNN (TSFFCNN). The proposed 1D-CNN and 2D-CNN parallel multichannel structures extracted the in-depth features and employed a feature fusion strategy to merge them in order to obtain a more reliable fault diagnosis [17].

However, most of the above research relied on the failure test bench in a laboratory environment, which can be operated via human modulation to generate a large number of fault data. A primary mine fan is normally required to work for a long time at coal mine industrial sites. Since the working environment is particular and dangerous, when a fan fails, it is stopped for maintenance immediately, making it challenging to collect fault operation data of the fan under actual working conditions. Moreover, most coal mining enterprises adopt a preventive periodic maintenance program to maintain and manage their primary mine fan, causing difficulties in the collection of fan fault operation data. For these reasons, the accumulation period of the primary mine fan’s rolling bearing fault data is long and time-consuming, and the attainable fault data are few in number and incomplete in type. Accordingly, the monitoring process results in a considerable number of normal data and very few fault data [18,19]. Thus, there is a severe sample imbalance in the operational monitoring data for the rolling bearings of primary mine fans. Using unbalanced samples for fault diagnosis increases a model’s sensitivity to majority class samples (i.e., normal data samples) and causes the classification boundary of the model classifier to be biased toward majority class samples. The minority class samples (i.e., fault data samples) are often considered the noise of the majority class samples, making their identification difficult and affecting the fault diagnosis accuracy, thus causing overfitting or misdiagnosis phenomena.

In order to promote the diagnostic model’s efficiency under the sample imbalance condition, it is necessary to change the data distribution and alleviate the sample imbalance. The most commonly used approaches are divided into two categories. One category is to improve the FD algorithm to enhance the classification precision of the minority classes. This is generally carried out by changing the loss function of the algorithm to devote suitable weights to the minority class samples. However, such methods may result in models that are overly dependent on loss function design. It may ignore other important features and information, which causes the model to perform poorly in the face of unknown data or changes in data distribution. Moreover, when assigning weights to minority samples, the weight settings need to be trial-and-error and optimized through manual experience. Finding the optimal weight setting requires considerable labor costs and extended experiment time. And with the change in working conditions, such methods need to be designed and adjusted according to the specific data and problems for the loss function and weight settings when used for different datasets, which increases the complexity of practical applications. Thus, such methods suffer from poor robustness, data dependence, and difficulties in setting weights when solving the sample imbalance problem. This leads to these methods providing limited diagnostic performance improvement, and it can be difficult to obtain the optimal weights, which does not completely solve the impact caused by the sample imbalance problem. The other category is to reduce the imbalance at the data level, mainly through sample generation methods to generate more minority class samples to achieve data balance and effectively alleviate the sample imbalance problem. SMOTE, ADASYN, RSMOTE, and other algorithms [20,21,22] are more widely used methods in this area. With the development of DL approaches, variational autoencoders (VAEs) and generative adversarial networks (GANs) are also extensively utilized for sample generation.

Wang et al. integrated the variational self-encoder theory with generative adversarial networks and designed an FD method using improved generative adversarial networks [23]. Zhao et al. established an improved GAN to enhance FD efficiency in unbalanced data and introduced a self-encoder-based approach for estimating the similarity of the generated samples [24]. Cabrera et al. proposed a GAN-based approach for the FD of reciprocating machines under data imbalances [25]. Zhou et al. designed generators and discriminators for GANs to produce more discriminative fault samples using a global optimization approach [19]. Shao et al. established a structure using an auxiliary classifier GAN (ACGAN) to learn and produce realistic 1D raw data from mechanical sensor signals. They introduced certain evaluation methods to assess the generated samples’ quality to evaluate the generated models’ performance [26]. Luo et al. combined DCGAN and CGAN to propose an imbalance FD approach using a conditional deep convolutional generative adversarial network (C-DCGAN) generative model [27]. Li et al. presented a Wasserstein GAN by adding an auxiliary classifier, which is able to generate highly similar samples for a few classes using an unbalanced training set and effectively improve fault diagnosis accuracy [28]. Huang et al. presented an improved labeled noise robust auxiliary classifier generative adversarial network (rAC-GAN) to produce a large number of fault data, achieving the features of real-sample probability distribution through limited data [29].

Although all the above studies have attained some results, some deficiencies still exist. First, these studies employ a modified sample generation method based on GANs, which improves the sample generation ability. However, an optimal evaluation index is required to determine whether the loss function converges. Moreover, training instability and gradient disappearance problems still exist [30]. The experimental data employed in these methods still result from the fault simulation test bench in the laboratory environment, and they require real operating data from actual working conditions for experimental verification, which lacks a certain level of applicability. The above issues may degrade the diagnosis accuracy of rolling bearing faults under unbalanced sample conditions.

The denoising diffusion probabilistic model (DDPM) [31] is a flexible and fast processing data generation model. Due to its powerful ability to generate samples, it has been extensively utilized in several applications like computer vision and natural language processing [32,33,34,35]. Compared with GAN, DDPM has major differences in terms of sample generation method, training method, and training stability. Specifically, GAN is a generative model based on adversarial training, where generating samples is accomplished through random sampling from the latent space and mapping using a generator. A generator and a discriminator are also required for adversarial training to improve the quality of the generated samples. DDPM is a generative model based on probability density estimation, which uses random perturbations to estimate the probability density function of the data. DDPM generates samples by performing a series of random perturbations on the probability density function of the data, thus achieving sample generation step by step, and the generated samples usually exhibit continuity and smoothness. Thus, the sample discontinuity problem of GANs due to sampling from potential space for generating samples is avoided. At the same time, DDPM is able to capture data distribution features more comprehensively, avoiding the model collapse problem that may occur in GANs.

During training, the generator and discriminator of the GAN are trained using an adversarial loss function with the goal of minimizing the difference between the generated and real samples. Unlike traditional optimization problems, GAN does not have a clear convergence criterion during adversarial training. It usually indirectly assesses whether the loss function converges by observing the quality of the generated samples, the accuracy of the discriminator, and the value of the loss function. Thus, the training process of GAN is unstable and may have problems, such as pattern crashes and nonconvergence of training. Compared with GAN, DDPM is trained using a specific loss function (log-likelihood function) to fit the probability density of the data without the need for adversarial training. This makes the training of DDPM more stable and easier to control as well as making the training process smoother and the possibility of training instability and gradient disappearance problems relatively small. DDPM can also use the log-likelihood value, loss function value, sample reconstruction error, and other evaluation indicators to determine the convergence of the loss function and optimize and adjust the model to improve the performance and effect of DDPM generation. Therefore, DDPM is more suitable for the generation of high-dimensional data and multimodal data.

Meanwhile, many researchers have shown that converting vibration signals into images is an effective FD method [36,37,38]. Compared with one-dimensional vibration signals, two-dimensional images can characterize richer feature information. Among the current commonly used image conversion methods, the grayscale image conversion method is too simple, and some fault features in the vibration signals may be lost during the conversion process. Although the most commonly used time-frequency image conversion method can effectively characterize vibration signals, there are deficiencies in the principle of the method. Among them, the linear time-frequency analysis method has limited time-frequency resolution, and the nonlinear time-frequency analysis method is subject to intrinsic cross-term interference. In comparison, the symmetrized dot pattern (SDP) image conversion method can map the sampling points in the vibration signal as polar diameters in polar coordinates and the adjacent sampling points as polar angles, thus converting a one-dimensional vibration signal into a two-dimensional SDP image consisting of mirror symmetry points in polar coordinates. The SDP method can better characterize the change in amplitude and frequency of time-series signals through a variety of shape features, with strong signal characterization and high differentiation between different types of vibration images.

Therefore, the current study presents an intelligent FD approach that is suitable for rolling bearings of a primary mine fan under sample imbalance conditions. First, the method converts the RB vibration signal into a distinctive SDP image via the SDP technique. Then, the DDPM method generates image samples with similar characteristics as the real minority image samples to expand the imbalanced dataset in order to achieve sample balance. Finally, a CNN is trained as an FD model to identify the rolling bearing condition. In order to assess the effectiveness of the presented approach, experimental validation was conducted via a conventional rolling bearing dataset and primary mine fan rolling bearing monitoring data under actual working situations.

The essential novelties of the present work are as follows:

(1) To illustrate the original vibration signal’s fault characteristics in a strong noise environment and improve the FD performance, the SDP approach converts the 1D vibration signal into a simple and intuitive 2D SDP image with apparent features.
(2) The DDPM method generates image samples with characteristics similar to the minority class fault image samples, which complements the minority class fault samples in the dataset and effectively reduces the imbalance.
(3) Combining the powerful sample generation capability of DDPM with the advantages of CNN in image processing, the current work introduces an FD approach for the rolling bearings of primary mine fans. The illustrated approach is able to resolve the problem of insufficient fault diagnosis accuracy caused by the sample imbalance of rolling bearing faults in primary mine fans under actual working conditions. Moreover, the approach is robust and can attain satisfactory diagnostic efficiency on the conventional rolling bearing dataset and the primary mine fan rolling bearing monitoring data with substantial noise interference under actual working conditions.

The remainder of the current paper is arranged as follows: Section 2 describes the basic theory of the approach employed in the current paper. The whole structure of the presented diagnostic method and the diagnostic steps are described in Section 3. The efficiency of the presented approach is experimentally evaluated in Section 4 using two bearing datasets. Section 5 summarizes the paper and provides directions for future work.

## 2. Theory

This section introduces the fundamental theory of SDP, DDPM, and CNN methods used in this paper.

### 2.1. SDP Approach

A symmetrized dot pattern (SDP) can describe the time-series signals’ amplitude and frequency variations and can be applied to visualize speech signals [39,40]. The SDP method can directly transform the original vibration signal, which is nonsmooth and non-Gaussian, into an SDP image that comprises mirror symmetry points in polar coordinates without a time-frequency transformation. This provides simple, convenient, and quick computation.

**Figure 2:**
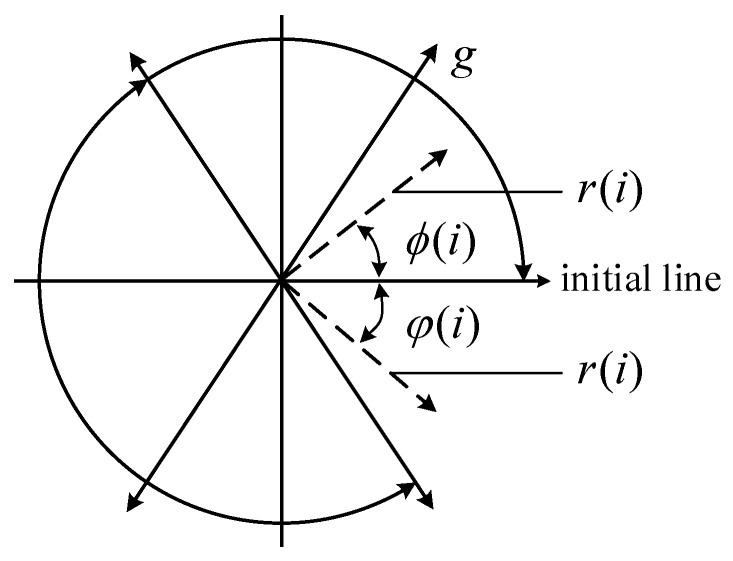
SDP schematic.

The vibration signal is a 1D time-series signal. When the vibration signal is transformed into an image using the SDP method, its amplitude and frequency variations can be reflected by the shape characteristics, geometric center, curvature, concentration area of points, and other elements of the SDP image, which can better describe the change features of the vibration signal. As shown in Figure 2, for the time-series $X=\left\{x_{1},x_{2},\ldots ,x_{i},\ldots ,x_{n}\right\}$, any point i in X can be converted into a point in the polar coordinate space $P\left(r\left(i\right),\phi \left(i\right),\varphi \left(i\right)\right)$.

In Figure 2, $r\left(i\right)$ describes the radius of polar coordinates, and $\phi \left(i\right)$ and $\varphi \left(i\right)$ describe the angles of polar coordinates rotated counterclockwise and clockwise along with the initial line, respectively. A set of signals $\left(x_{i},x_{\left(i+L\right)}\right)$ can be converted into mirror symmetry point images in polar coordinates by changing the initial line’s rotation angle using the following conversion equation:

(1) $$r(i)=\frac{x_{i}-x_{\min}}{x_{\max}-x_{\min}}$$

(2) $$\phi (i)=\theta +\frac{x_{\left(i+L\right)}-x_{\min}}{x_{\max}-x_{\min}}g$$

(3) $$\varphi (i)=\theta -\frac{x_{\left(i+L\right)}-x_{\min}}{x_{\max}-x_{\min}}g$$

where $x_{\max}$ and $x_{\min}$ describe the sampled data’s maximum and minimum values, respectively; L describes the time interval (generally between 1 and 10); θ describes the initial line rotation angle (the value is 360 *m*/*n*, *m* = 1, …, *n*, where *n* is the number of symmetric mirror planes, typically 6); and g describes the angular amplification factor (usually a value less than θ).

### 2.2. Denoising Diffusion Probabilistic Models (DDPM)

**Figure 3:**
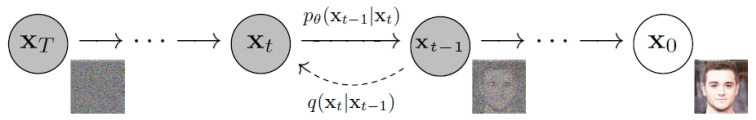
DDPM schematic.

With the continuous development of depth generation model research, the diffusion model has been extensively utilized in image generation. The concept of the diffusion model initially originated in 2015 [41]. After continuous development, Jonathan Ho et al. presented the DDPM in 2020 by improving the mathematical architecture based on the original diffusion model [31]. DDPM is an excellent image generation method that enables image generation under high-resolution conditions. The basic concept of DDPM is to destroy the distribution structure of the original data by continuously adding Gaussian noise and then performing reverse sampling to recover the original data structure from the inverse process. Figure 3 presents the schematic diagram of DDPM.

As shown in Figure 3, the DDPM model comprises two processes: forward-noise addition and reverse-noise removal.

In the forward-noise addition process, Gaussian noise is continuously added to the original clear data $x_{0}\sim q\left(x\right)$ according to a predetermined noise schedule (T-round) until the data distribution tends toward the standard Gaussian distribution, obtaining pure Gaussian noise data $x_{T}\sim N\left(0,Ι\right)$. The process of adding Gaussian noise can be expressed as follows:

(4) $$q(x_{t}|x_{t-1})=N(x_{t};\sqrt{1-\beta_{t}}x_{t-1},\beta_{t}Ι)$$

(5) $$q(x_{1:T}|x_{0})=\prod_{t=1}^{T}q(x_{t}|x_{t-1})$$

where T describes the number of rounds of added noise, ${\left\{\beta_{t}\in \left(0,1\right)\right\}}_{t=1}^{T}$ is a predetermined monotonically increasing control parameter that controls the size of each added noise, $N\left(\cdot \right)$ denotes the normal distribution, $\prod_{t=1}^{T}\left(\cdot \right)$ denotes the concatenated multiplication operation, and Ι is the variance of the Gaussian noise.

In the reverse denoising process, it is necessary to derive the conditional probability $q(x_{t-1}|x_{t})$ to restore the original data distribution $x_{0}\sim q\left(x\right)$ from the full standard Gaussian distribution noise $x_{T}\sim N\left(0,Ι\right)$. However, since this process requires the distribution of all possible images, the conditional probability $q(x_{t-1}|x_{t})$ cannot be directly calculated. According to a related study [42], if $q(x_{t}|x_{t-1})$ follows a Gaussian distribution, and $\beta_{t}$ is small enough, $q(x_{t-1}|x_{t})$ still follows a Gaussian distribution. Therefore, a deep learning model can be employed to fit this distribution, and the probability distribution $p_{\theta }(x_{t-1}|x_{t})$ obtained from the deep learning model can be employed instead of $q(x_{t-1}|x_{t})$. The fitting procedure can be described as follows:

(6) $$p_{\theta }(X_{0:T})=p(x_{T})\prod_{t=1}^{T}p_{\theta }(x_{t-1}|x_{t})$$

(7) $$p_{\theta }(x_{t-1}|x_{t})=N(x_{t-1};\mu_{\theta }(x_{t},t),\sum_{\theta }\left(x_{t},t\right))$$

Given the condition $x_{0}$, using the Bayesian equation and substituting the Gaussian distribution expression, the following relation can be obtained:

(8) $$q(x_{t-1}|x_{t},x_{0})=N(x_{t-1};{\tilde{\mu }}_{t}(x_{t},x_{0}),{\tilde{\beta }}_{t}Ι)$$

The variance ${\tilde{\beta }}_{t}$ and the mean ${\tilde{\mu }}_{t}\left(x_{t},x_{0}\right)$ are obtained as follows:

(9) $${\tilde{\beta }}_{t}=\frac{1-{\bar{\alpha }}_{t-1}}{1-{\bar{\alpha }}_{t}}\beta_{t}$$

(10) $${\tilde{\mu }}_{t}(x_{t},x_{0})=\frac{\sqrt{{\bar{\alpha }}_{t-1}}\beta_{t}}{1-{\bar{\alpha }}_{t}}x_{0}+\frac{\sqrt{\alpha_{t}}(1-{\bar{\alpha }}_{t-1})}{1-{\bar{\alpha }}_{t}}x_{t}$$

When using DDPM for image generation, the noise signal is first sampled from a known distribution, and the distribution of complex images is then sampled via the multistep denoising of the denoising network. The goal of training the DDPM is to minimize the error between the network’s predicted and actual noise distributions at time t. Therefore, it is necessary to maximize the log-likelihood of the model’s predicted distribution to the actual distribution of the data when training the DDPM, i.e., to optimize the $p_{\theta }\left(x_{0}\right)$ cross-entropy under $x_{0}∼q\left(x_{0}\right)$. The following equation describes the maximization objective:

(11) $$L=E_{q(x_{0})}\left[-\log p_{\theta }(x_{0})\right]$$

Equation (11) can be simplified using the variational method to describe the model’s training objective with the following objective function at any t moment:

(12) $$\begin{array}{cc} L_{t}^{simple} & =E_{x_{0},{\bar{z}}_{t}}[∥{\bar{z}}_{t}-z_{\theta }(x_{t},t){∥}^{2}]\\ & =E_{x_{0},{\bar{z}}_{t}}[∥{\bar{z}}_{t}-z_{\theta }(\sqrt{{\bar{\alpha }}_{t}}x_{0}+\sqrt{1-{\bar{\alpha }}_{t}}{\bar{z}}_{t},t){∥}^{2}] \end{array}$$

Accordingly, the training procedure of DDPM can be described as follows:

(1) Obtain the input $x_{0}∼q\left(x_{0}\right)$ and random samples t from 1∼T;
(2) Acquire a sample of the noise ${\bar{z}}_{t}\sim N\left(0,Ι\right)$ from a standard Gaussian distribution;
(3) Minimize $∥{\bar{z}}_{t}-z_{\theta }\left(\sqrt{{\bar{\alpha }}_{t}}x_{0}+\sqrt{1-{\bar{\alpha }}_{t}}{\bar{z}}_{t},t\right)∥$.

### 2.3. Convolutional Neural Network

**Figure 4:**
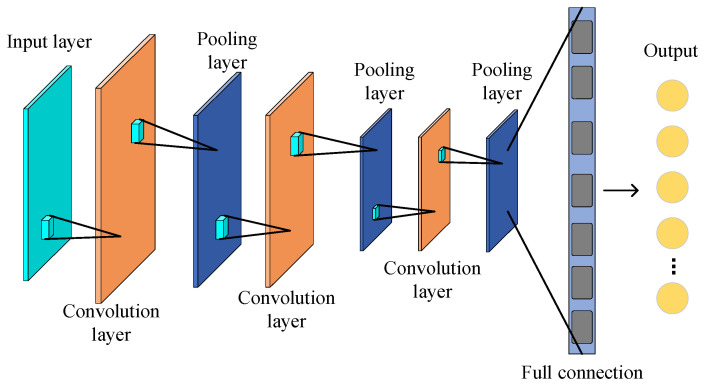
CNN structure diagram.

A convolutional neural network (CNN) is a typical feed-forward neural network with significant advantages in 2D image processing. A CNN is based on the extraction of the features of the input data via the construction of multiple filters layer by layer. As shown in Figure 4, a typical CNN usually comprises convolutional, pooling, and fully connected layers, where the convolutional and pooling layers are alternately stacked.

In CNNs, the convolutional layer operates by convolving multiple local filters with the original input data through a convolutional kernel to extract local features and output a feature map. The number of output feature maps is the depth of the convolutional layer. After completing the convolutional operation, the activation function is generally utilized to achieve the nonlinear transformation of the output of the convolutional layer to accelerate the convergence of a CNN.

The pooling layer usually utilizes a specific statistical feature of the adjacent output at a location to replace the network’s output at that location, which is essentially the scaled mapping of the previous layer’s data graph. The pooling layer is able to efficiently alleviate the data dimension and network parameters, accelerate the CNN calculations, and avoid network overfitting.

The fully connected layer constructs a feature vector by sequentially expanding all the extracted feature maps. Its primary function is to integrate the information from the previous layers.

A CNN provides some advantages, such as sparse connectivity and weight sharing. Sparse connectivity is able to alleviate the number of training parameters, and weight sharing can efficiently prevent the algorithm’s overfitting. Meanwhile, the batch normalization (BN) layer and dropout technique are usually utilized in CNNs. Of the two, the BN layer performs the normalization operation on each batch of input data, which overcomes any variation in the data distribution in the middle layer, solves the unstable training phenomenon in the deep layer network, and increases the CNN’s convergence rate. The dropout technique reduces the overfitting phenomenon of CNN models by setting parameters to deactivate the neurons in the fully connected layer with a certain probability.

The CNN’s training procedure comprises forward and backward propagation. Forward propagation aims to input the sample data into the CNN and obtain the network output. Backpropagation computes the error between the network output and the actual value from the loss function and propagates the error backward. The whole convolutional neural network is trained to minimize the loss function.

## 3. Fault Diagnosis Method

**Figure 5:**
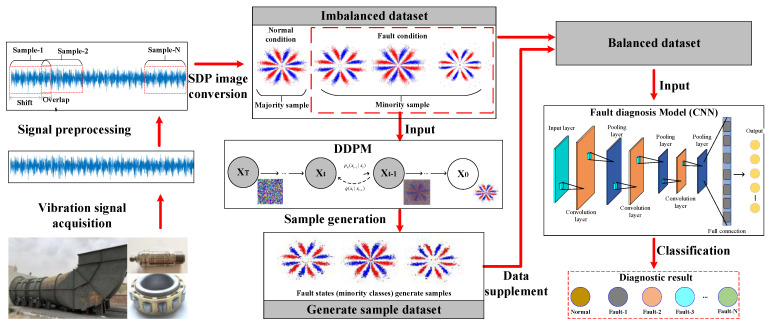
Flowchart of fault diagnosis method.

The current paper proposes an intelligent FD approach for the rolling bearings of primary mine fans under sample imbalance conditions based on the SDP technique, denoising diffusion probabilistic models (DDPMs), and convolutional neural networks (CNNs). Figure 5 shows the method’s flowchart.

The presented FD approach combines the sample generation ability of DDPM with the feature extraction capability of CNN to effectively solve the sample imbalance and insufficient fault diagnosis performance problems induced by insufficient fault samples of the rolling bearings in a primary mine fan. The diagnostic process of the presented approach can be described in the subsequent stages:

(1) The vibration signals are acquired from rolling bearings via sensors and data acquisition instruments.
(2) The overlapping sampling approach is employed to preprocess the vibration signal and divide it into several fixed-length samples. The 1D vibration signal is then converted into a 2D SDP image using the SDP approach and taken as the input sample for the FD model. The normal and fault-state samples are the majority and minority class samples, respectively.
(3) The input samples are categorized into imbalanced training datasets and balanced test datasets.
(4) Training samples of each minority class (fault state) are input into the DDPM individually to generate more samples of generated images with similar feature distributions as the real images.
(5) The generated minority class (fault state) samples are added to the unbalanced training dataset to the sample expansion of the minority class samples to balance the training dataset sample. A balanced training dataset containing the generated and original images is then utilized as the input for training the CNN-based FD model so that the model can identify various fault-state features of the bearing.
(6) The trained FD model is evaluated using the test dataset, and the efficiency of the presented approach is assessed for the final FD accuracy.

In this paper, the core process of the proposed rolling bearing fault diagnosis method in primary mine fans includes three steps, namely SDP image conversion, DDPM image generation, and CNN fault diagnosis model training. The method makes full use of the advantages of SDP technology and DDPM, combined with the feature extraction capability of CNN to solve the problems of difficult-to-train fault diagnosis models, insufficient fault diagnosis accuracy, and easy overfitting or misdiagnosis due to sample imbalance. And it is capable of accurately detecting rolling bearing faults in primary mine fans under sample imbalance conditions. Of these, SDP images are able to highlight the fault characteristics of the bearing vibration signals so that they can be easily recognized and extracted, which helps to improve the fault diagnosis performance. The DDPM is used to generate image samples with similar characteristics to the minority category of fault image samples, which effectively expands the imbalance dataset and reduces the degree of data imbalance. In turn, the fault diagnosis model is better able to learn the features of the minority class fault samples and improve the accuracy of fault detection. Finally, the advantages of CNN in image processing and feature extraction tasks are fully utilized to identify and classify the faults of the rolling bearings in primary mine fans. The method proposed in this paper provides a novel and effective solution for the intelligent fault diagnosis of rolling bearings of primary mine fans. Moreover, the method is robust and can provide a reference for other similar problems in engineering practice.

## 4. Experimental Verification

The presented approach aims to resolve the sample imbalance issue in the fault diagnosis of the primary mine fan rolling bearings. In order to assess the efficiency of the developed approach, the current paper employs the conventional rolling bearing fault dataset collected in the laboratory and the rolling bearing dataset of a primary mine fan in actual working conditions for experimental validation. All experiments were performed on a computer with the operating system Ubuntu 18.04LTS, CPU Intel(R) Xeon(R) 3204, and GPU Nvidia GeForce GTX 3080. Vibration signal processing and SDP image conversion were performed using MATLAB 2017a. The programming structure for the DL method was PyTorch, and the programming language was Python.

### 4.1. Experimental Verification Based on Conventional Rolling Bearing Fault Data

**Figure 6:**
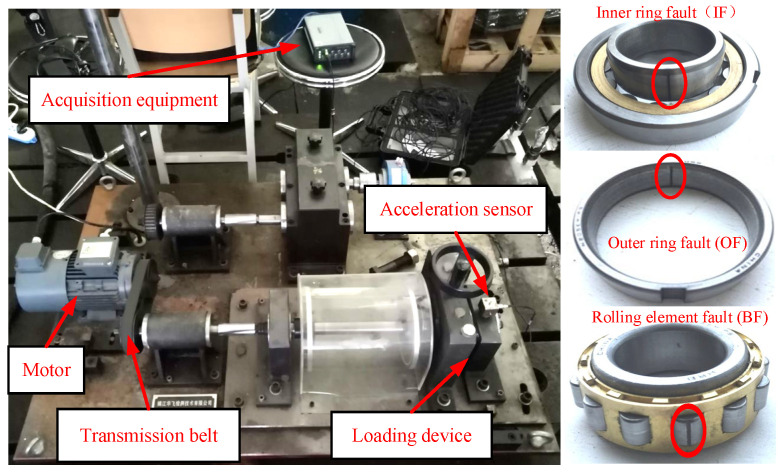
QPZZ-II rotating machinery fault test bench.

In order to evaluate the efficiency of the developed approach, the experiments in the current section employed the QPZZ-II rotating machinery failure test bench dataset for experimental validation. The rolling bearing’s simulated failure part was utilized in the experiment, as presented in Figure 6. The test bench predominantly encompassed the motor, drive belt, loading device, acceleration sensor, and test bearing. The test bearing was mounted inside the loading device, and the acceleration sensor was mounted above the bearing housing.

The motor’s rotation speed was 1500 r/min, and the load was 0 HP. The wire-cutting technique was utilized to set up a single point of failure on the inner ring, outer ring, and rolling element of the tested bearing. The test bench included an INV9824 accelerometer and an INV3062C collector to acquire bearing vibration signals, and the bearing vibration data’s sampling frequency was 10.24 KHz. First, the normal bearing’s vibration acceleration signal was acquired, and then three kinds of fault rolling bearings (inner ring, outer ring, and rolling-element faults) were mounted to the test bench for fault signal collection.

#### 4.1.1. Evaluation and Analysis of Generated Images

After preprocessing the collected vibration data, the overlapping sampling technique was utilized to divide the four operating states’ vibration signals into samples, each containing 2048 sampling points. To perform sample segmentation, the first step was to set the size of the sampling window to 2048. The size of the sampling window determined the length of each sample intercept from the vibration signal. Then, the sampling step size was set to 300, which determined how far the sampling window moved through the vibration signal, allowing it to intercept the next sample. A series of overlapping sets of vibration samples were obtained using the overlapping sampling method. Finally, the vibration samples with the same window length were converted to SDP images and resized to 64 × 64 using the conversion Equations (1)–(3) in Section 2.

As the quality of SDP images directly depends on the parameters θ, L, and g, in this study, we first conducted experimental research on the parameter selection of SDP. Numerous studies have indicated that SDP images’ symmetry and shape features are more prominent when θ is chosen at 60°. A reasonable selection of parameters *g* and *L* can promote the image quality and strengthen the distinctions between signals, allowing more apparent differentiation among various vibration signals [43,44]. Therefore, using the image correlation coefficient approach, the current paper verifies the correlation between images of different fault categories. For two m×n images, the correlation coefficient R can be described as follows:

(13) $$R(A,B)=\frac{\sum_{m}\sum_{n}\left(A_{mn}-\bar{A}\right)(B_{mn}-\bar{B})}{\sqrt{\left[\sum_{m}\sum_{n}{\left(A_{mn}-\bar{A}\right)}^{2}\right]\left[\sum_{m}\sum_{n}{\left(B_{mn}-\bar{B}\right)}^{2}\right]}}$$

where *A* and *B* describe the images’ two-dimensional gray matrices. The smaller the correlation coefficient *R* of different images, the greater the difference between images. In order to better choose the optimum values of g and L to detect the SDP images of various fault states, the sum of the correlation coefficients between the SDP images of the four states is utilized as the image assessment metric in this paper, which is described as follows:

(14) $$R_{sum}=\sum_{\begin{array}{l} i\neq j\\ i<j \end{array}}R(A_{i},B_{j})(i=1,\cdots ,k-1;j=2,\cdots ,k;k=4)$$

**Figure 7:**
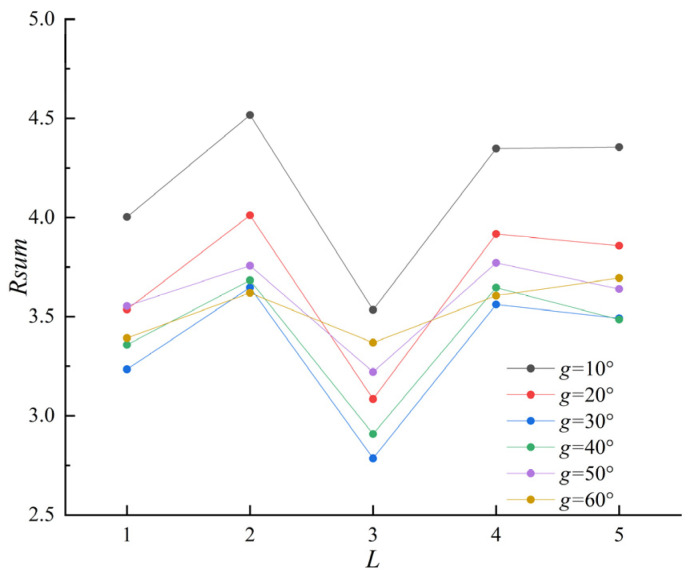
Correlation between SDP images for different parameter values.

The values of L are set from 1 to 5, and the values of g are set from 10° to 60° in steps of 1 and 10°, respectively. Figure 7 shows the sum of the correlation coefficients of the four operating SDP images. The minimum value of the sum of correlation coefficients Rsum is obtained for L = 3 and g = 30°. At this point, the correlation between the SDP images of various states is the smallest, and the recognizability is the highest.

**Figure 8:**
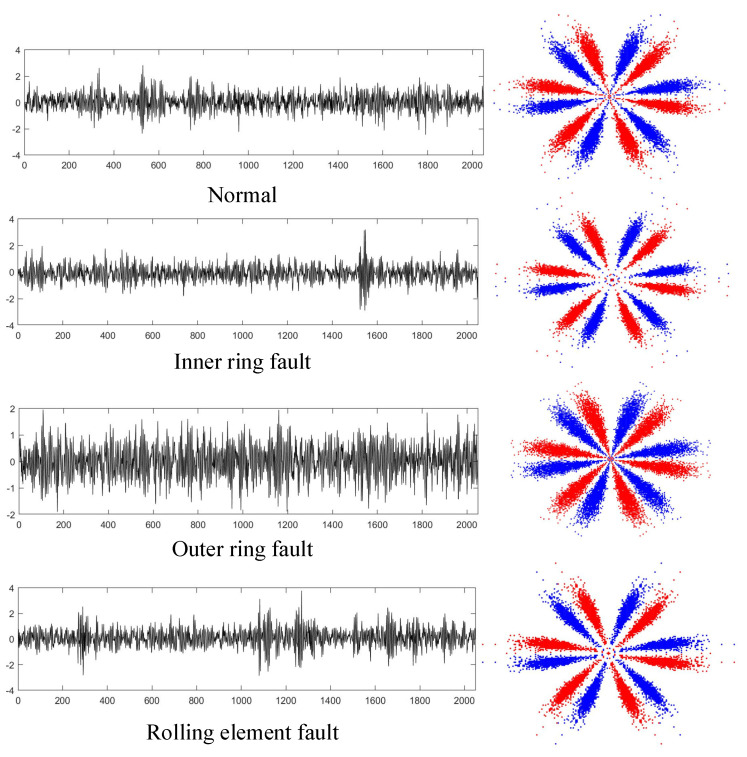
Time-domain and SDP graphs for the four operating states of rolling bearings.

After selecting the optimal parameters, the SDP approach was utilized to transform all vibration signals into two-dimensional SDP images. The time-domain graphs of the four operating state samples of the rolling bearing and the converted SDP images are shown in Figure 8.

In order to conduct research on rolling bearing fault diagnosis under sample imbalance conditions, a sample set for training and testing the diagnostic model based on SDP images was constructed. Therefore, 1500 samples were randomly chosen from the normal samples as training samples, and 200 samples were randomly chosen as the test samples. The other three types of faulty samples were randomly chosen from 1000 samples as training samples and 200 samples as test samples. As shown in Table 1, an unbalanced dataset was eventually constructed artificially for the experiment.

The normal-state images in the dataset were chosen as majority class samples, and the other three fault-state images were chosen as minority class samples. After obtaining the SDP images of different operating states of rolling bearings, three minority sample images of each class were utilized as the input for training the DDPM and supplementing the imbalance sample set using the DDPM-generated images.

**Figure 9:**
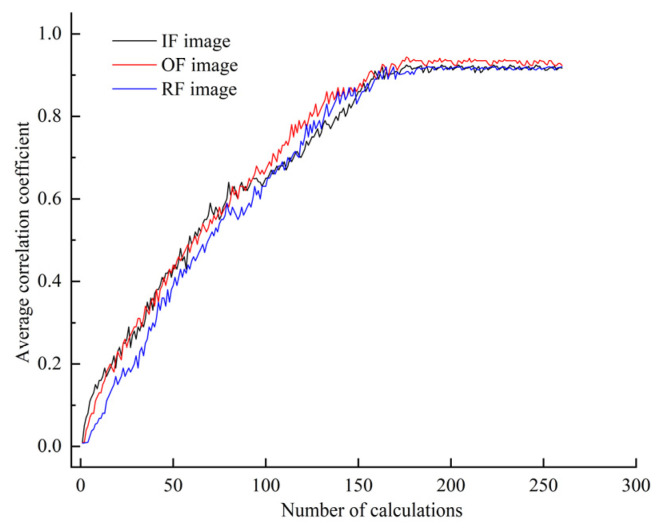
Average correlation coefficients between generated and real images for different faults.

In order to validate the efficiency of the DDPM approach, the quality of the produced samples should be evaluated, and their impact on FD under sample imbalance conditions should be studied. The DDPM was modeled using the U-Net, a U-shaped network framework comprising an encoder, a decoder, and cross-layer connections (residual links) between the encoder and decoder. The U-Net model first achieves downsampling operations on the input through the encoder and then accomplishes upsampling operations using the decoder, with cross-layer connections employed to splice features between the encoder and decoder. The specific model parameter settings can be found in [31]. In DDPM, a T = 1000 and batch_size = 16 were chosen, and the Adam optimizer was utilized for optimizing the neural network. In the training process of DDPM, the image samples were first input into the DDPM, and a noise path was formed by propagating the noise gradually to each reversible layer through forward propagation. The conditional probability between the input image and its denoised output was then computed at the ascent step of each reversible layer, and the denoising effectiveness of the model for a given input image was measured through the conditional probability. Next, the conditional probability loss function of the DDPM was calculated, and the gradient of the loss function with respect to the model parameters was computed via the backpropagation algorithm. Then, an optimization algorithm was used to update the parameters of the DDPM according to the calculated gradient so that the loss function was gradually reduced. Finally, the process of forward propagation, calculating conditional probabilities, backpropagation, and parameter updating was repeated until a preset number of training rounds was reached or certain convergence conditions were achieved. However, the training rounds of DDPM are generally set according to manual experience and without a theoretical basis. Too many training rounds result in wasted computational resources and experiment time, while too few training rounds result in poor-quality generated images. Moreover, all of the generated images cannot be expanded and enhanced for the imbalanced dataset in the training results of DDPM. Therefore, the correlation coefficient between each generated image and the real image in the current batch was calculated every 25 rounds of training during the DDPM training process. The average of all correlation coefficients was then calculated, and the result was employed as an evaluation metric to determine whether the DDPM-generated image qualities are up to standard and can be added to the imbalanced dataset for data enhancement. Figure 9 shows the results.

As presented in Figure 9, the change in the average correlation coefficient of the images with different faults begins to level off after 180 calculations when the training round of DDPM is 4500. Therefore, the DDPM training rounds were set to 5000 (epochs = 5000), as it is considered that the generated images of the DDPM after 5000 training rounds can be effectively utilized to expand the imbalanced dataset. The training weights were saved after the training, and DDPM employed the weights for image generation in subsequent experiments.

**Figure 10:**
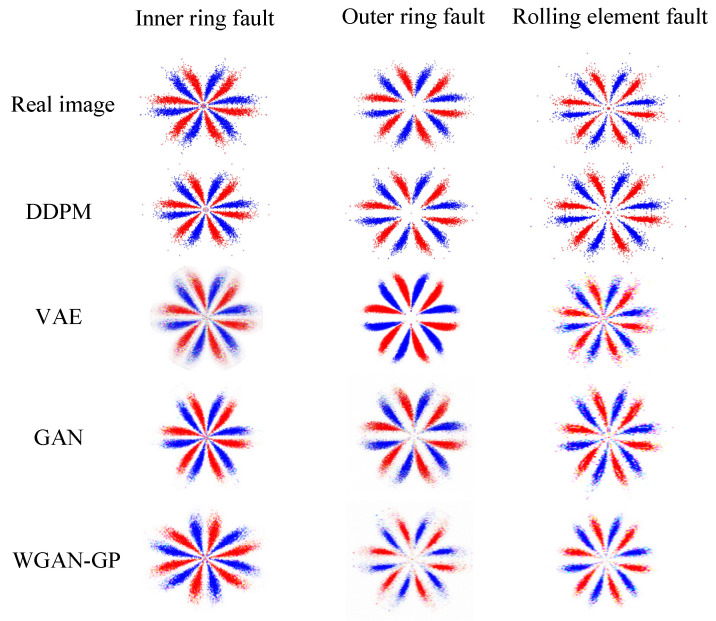
Comparison of the real image with different types of generated images.

Three minority class images were separately adopted as the input, and the trained DDPM was employed for image sample generation. These were compared with the samples generated via other common generation methods, such as VAE, GAN, and WGAN-GP, as presented in Figure 10. In this case, a fully connected network was employed to construct the encoder and decoder of VAE. The encoder and decoder structures were 4096-1024-512-64 and 20-64-512-1024-4096, respectively. The Adam optimizer was utilized for training, with a learning rate of 0.001. The discriminators and generators of GAN and WGAN-GP both employed convolutional neural networks to construct the network, and the Adam optimizer was utilized for optimizing the neural network, with a learning rate of 0.0002. A BN layer was added to the generator to normalize the data and prevent the model from overfitting, and ReLU was adopted as the activation function after the BN layer. Tanh and LeakyReLU were used as the activation functions in the output layer and the discriminator, respectively.

**Figure 11:**
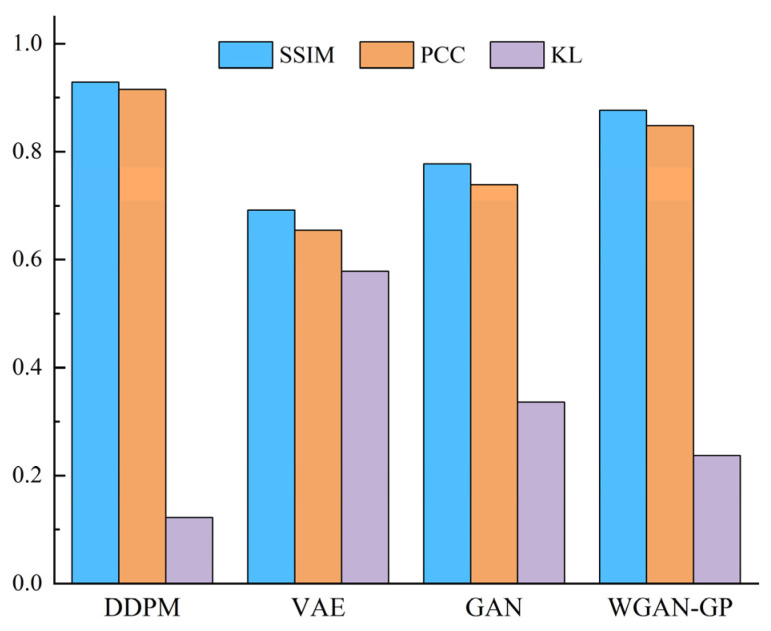
Comparison of the quality of generated images based on evaluation metrics.

Figure 10 shows that the DDPM-generated images are intuitively more similar to the actual image, specifically in that the shape features and geometric center of the SDP image are more similar to the actual image. This demonstrates the superiority of the DDPM-generated images’ quality and image similarity to other generation approaches. In order to quantitatively assess the quality of the produced images, ten produced images were selected from the generated images of each method. Then, the structural similarity (SSIM), Pearson’s correlation coefficient (PCC), and KL divergence (KL) between all the produced images and the relative real images were calculated, and the mean value of the above indexes was chosen as the assessment basis for the quality of the produced images, as presented in Figure 11. The SSIM determines the similarity of two images whose value changes from 0 to 1. The larger the SSIM value, the more similar the image is. PCC is an indicator that reflects the correlation between different distributions and takes a value between 0 and 1. The larger the PCC value, the more similar the distribution. KL measures the difference between distributions and takes a value from 0 to ∞; the smaller the KL value, the more similar the distribution.

Figure 11 shows that the presented DDPM approach generates images superior to other generation approaches in all evaluation metrics. It shows that the images produced using the DDPM approach have high quality, a similar appearance between the produced and real images, and similar feature distribution. Therefore, using DDPM to generate a few class fault images to complement the imbalance dataset is a feasible and effective approach.

**Figure 12:**
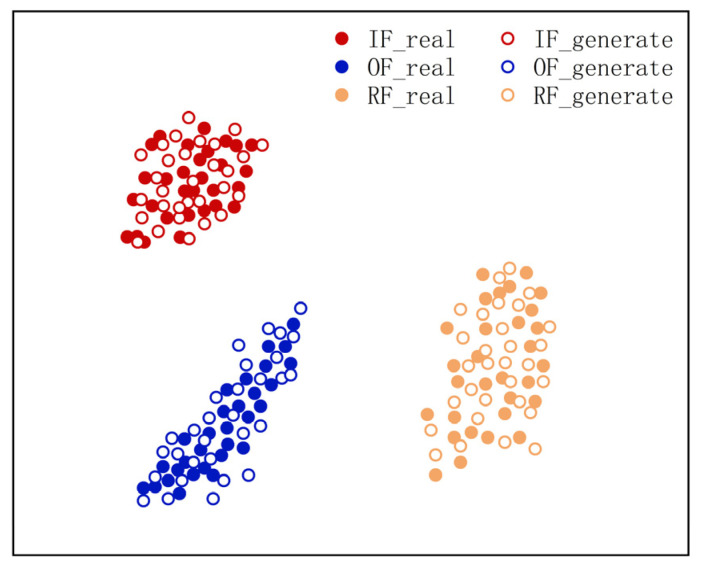
Feature visualization of the produced and real images.

In order to further describe the advantages of the DDPM approach for generating images, the t-SNE method [45] was used to visualize the partial DDPM-generated and real images of three fault types in a reduced dimension, and the visualization results of the sample distribution verified the similarity between the produced and actual images. Figure 12 shows the results.

As presented in Figure 12, the produced and actual images can be aggregated into one class, and their feature distributions are in the same area and do not overlap. This indicates that the DDPM-generated image samples have high similarity to the real image samples without identical feature distributions. It also illustrates that DDPM can effectively fit the feature distribution of real images and that the produced images have sample diversity and high similarity.

In summary, DDPM can generate high-quality image samples. Compared with other generation approaches, the DDPM-produced samples have a higher similarity to the real samples and are better at enhancing and complementing the unbalanced sample set.

#### 4.1.2. Bearing Fault Diagnosis Research under Multiple Sample Imbalance Conditions

The efficiency of deep neural network-based FD approaches depends on the number of training samples. Under the sample imbalance condition, the sample generation method can effectively solve the diagnostic model’s insufficient precision due to the lack of a few classes of training samples. However, the produced samples are derived from the real ones and can only complement, but not completely replace, the real samples for a few classes of real samples.

In this study, we conducted different experiments on the imbalance rate of a few classes of samples to assess the influence of the complementary number of produced samples on the fault diagnosis accuracy. According to the multiple imbalance rate settings, in the experiments, a few classes (fault states) of samples were first generated using the DDPM method, and then data augmentation was performed on the imbalance sample set, i.e., the produced samples were used to supplement the real samples and finally obtain a mixed sample set, as described in Table 2.

In all the mixed sample sets, data augmentation was performed only in the training set, and the test set comprised only the original real samples to ensure consistent results. Moreover, the number of majority class (normal state) samples employed for training was not fixed and was the same as the number of expanded minority class ones.

The presented CNN-based fault diagnosis model was trained using the above dataset, and the trained model was evaluated through the test set. The CNN diagnosis model comprised five alternately connected convolutional and pooling layers, two fully connected layers, and a SoftMax classifier. The size of the convolutional kernel was 3 × 3, the number of convolutional kernels was 16, 32, 32, 64, and 128; for the pooling layer, 2 × 2 maximum pooling was used; the activation function was ReLU; and the number of nodes in the fully connected layer was 1024 and 256. In order to avoid the network from overfitting, a BN layer was added after the first convolutional layer, and a dropout operation was added after the fully connected layer, where the value was set to 0.25. The model was trained with a batch_size = 64 and an epoch = 500, and the learning rate was chosen as 0.001. The model utilized a zero-padding method to increase the dimensionality so that the feature map size remained the same before and after convolution. In the training process of CNN, the weights and biases of the model first had to be initialized using small random numbers. Then, the data were passed from the input layer all the way to the output layer through a forward propagation process. During forward propagation, CNN extracted image features through a series of convolution operations, activation functions, and pooling operations. After that, the output of the forward propagation was compared with the real label, and the value of the loss function was calculated. Next, CNN calculated the gradient of the loss function with respect to the model parameters via backpropagation, and the Adam optimization algorithm was used to update the model parameters so that the value of the loss function gradually would approach the optimal solution. Finally, the process of forward propagation, computation of losses, backpropagation, and parameter updating was repeated until a preset number of training rounds was reached, or certain convergence conditions were achieved.

**Figure 13:**
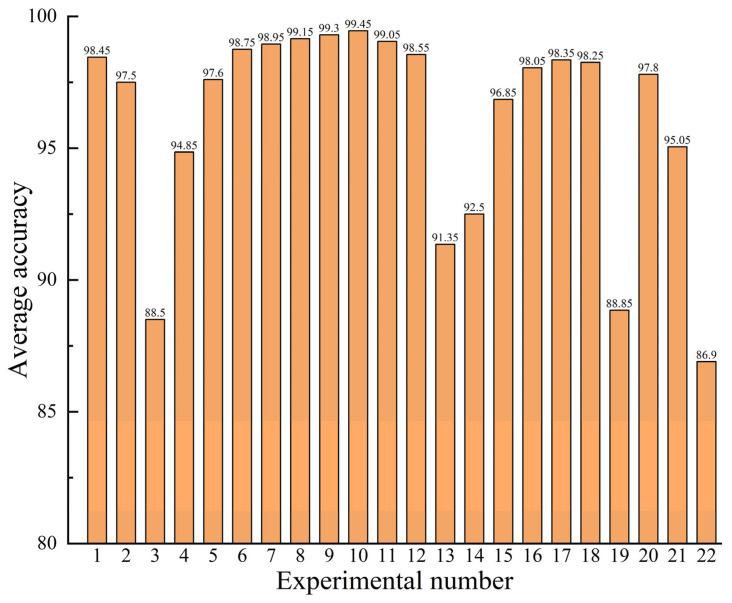
Comparison of fault diagnosis accuracy for multiple imbalance rate datasets.

In order to diminish the effect of chance errors, all datasets were tested 10 times, and their diagnostic efficiency was evaluated through the mean precision of the 10 test results. Table 2 and Figure 13 present the average accuracy of the FD model on all datasets.

Considering the above experiments, Experiments 1–3 only involved the original real samples, Experiments 4–19 were conducted using the generated samples for data augmentation, and Experiments 20–22 were conducted using only the generated samples for training the diagnostic model.

The analysis of the results of Experiments 1–3 and Experiments 20–22 indicates that the efficiency of the FD model trained on the produced samples only is lower than that using the real samples only. The reason for this is that the generated sample is extracted from the actual sample, which only supplements the real sample and cannot completely replace it. Moreover, it can be concluded from Experiments 3, 19, and 22 that the diagnostic accuracy is low for less than 500 training samples, indicating that the number of training samples considerably affects the diagnosis model’s precision.

The comparison of the results of Experiment 1 with Experiments 6–12 and Experiment 2 with Experiments 16–18 indicate that the diagnostic accuracy can be effectively improved by adding additional produced samples to the training sample set for sample enhancement in FD model training. When the ratio of the number of generated samples to the number of real samples is between 0.25 and 1, there is a considerable improvement in the diagnosis model’s accuracy. The best improvement in diagnostic precision can be attained when the number of generated samples is half the number of real ones. Data enhancement using generated samples for unbalanced datasets is an effective method when performing FD under sample imbalance situations, and the efficiency and feasibility of the presented approach are also illustrated.

However, the number of added generated samples is not as high as possible during sample augmentation. The results of the comparisons of Experiment 1 with Experiments 4–7 and Experiment 2 with Experiments 13–15 show that adding more generated samples than real samples cannot significantly affect the diagnostic model and enhance diagnostic efficiency. The accuracy of the diagnostic model is reduced when adding too many generated samples. This is because the generated samples contain other information about the real samples’ characteristics and generation errors. Excessive interference can degrade the diagnosis model’s efficiency, further illustrating that the generated samples cannot completely replace the real ones.

#### 4.1.3. Application of Sample Generation Method in Rolling Bearing Fault Diagnosis

In order to further assess the feasibility of adding produced samples to the imbalanced sample set for data augmentation in FD and the efficiency of the presented approach, the proposed method was validated via experiments.

In these experiments, different sample generation methods were first used to supplement the data of the unbalanced sample set shown in Table 1 and reach the sample equilibrium state. The optimal number of supplements was employed, i.e., 500 additional generated images were added to the image sample for each class of fault states to make the number of images in the minority class (fault state) equal to that in the majority class (normal state). Experimental validation was then performed using a mixed sample set, as presented in Table 3.

The above dataset was employed to train the CNN diagnosis model, while its architecture and parameter settings were kept unchanged. Meanwhile, to evaluate the efficiency of the presented approach, other diagnosis models, like the support vector machine (SVM), random forest (RF), and BP neural network, were trained based on the same dataset for comparison. The CNN diagnosis model used in this paper adopted the SDP image directly as the input, while the SDP image’s texture feature parameters were chosen as the inputs of other diagnosis models. The accuracy of different datasets in different fault diagnosis models was then evaluated using the test set. In order to alleviate the impact of chance errors, each method was tested 10 times, and their diagnostic efficiencies were evaluated through the mean precision of the 10 test results, as presented in Table 4.

The experimental results indicate that the classification precision of all fault diagnosis approaches on the mixed dataset with the addition of the produced samples is superior to the unbalanced dataset. The reason for this is that the hybrid dataset has more image samples that can be employed for training. This reveals that when performing FD under the sample imbalance condition, using the sample generation method to generate additional minority class samples for supplementing the imbalanced dataset can effectively increase the classification precision of the FD model and solve the low diagnosis precision problem caused by the lack of training samples.

Meanwhile, the test results of all the fault diagnosis models on the mixed sample set indicate that generating a few classes of samples for data supplementation using the DDPM method can improve the diagnosis classification precision compared with other generation methods. This reflects that the images generated via the DDPM method have better quality, with higher similarity to the real images and a better enhancement of the original unbalanced data. The DDPM-based sample generation method can be utilized as an efficient data enhancement method to promote the diagnostic precision and generalization efficiency of FD classifiers under unbalanced sample conditions.

The experimental results also indicate that the presented CNN FD model achieves the highest diagnostic precision on all datasets, which is because the CNN model is a deep neural network. Compared with the other three shallow networks, CNN is able to extract more and deeper characteristics to efficiently characterize the complicated mapping relationship between RB vibration signals and fault states. Another reason for this is that the input of CNN is an SDP image, which can be better characterized for rolling bearing faults. At the same time, the inputs of SVM, RF, and BP neural networks are the SDP images’ texture feature parameters, which are subject to uncertainty caused by human interference in the extraction procedure, and some critical fault features may be lost.

**Figure 14:**
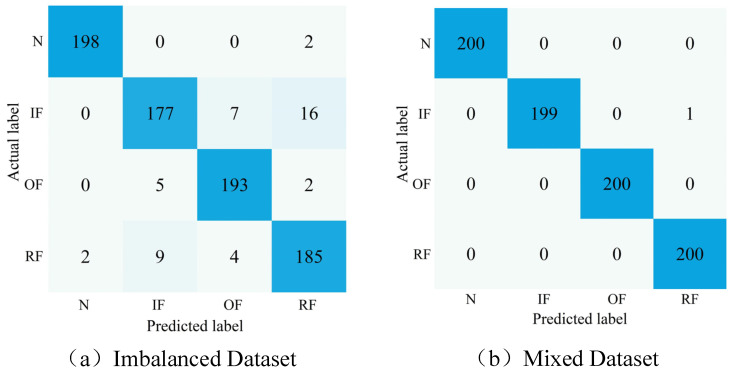
Classification confusion matrix for different datasets.

In order to further illustrate the efficiency of the developed approach, Figure 14 shows the confusion matrix for the first experiment when employing the CNN model for FD and the classification of the imbalanced and mixed datasets after sample enhancement using DDPM. The confusion matrix’s vertical coordinates describe the actual label of the classification, and the horizontal coordinates describe the predicted label. The confusion matrix’s main diagonal elements describe the number of truly classified samples in the current category.

The classification effect of the CNN FD model in a mixed dataset is considerably better than that on the unbalanced dataset. Enhancing the unbalanced dataset using DDPM-generated image samples can improve the diagnostic model’s classification precision in unbalanced situations. Meanwhile, the mentioned results also indicate that the FD method using the SDP images, DDPM, and CNN model presented in the current work can accurately classify rolling bearings’ different operating conditions in the sample imbalance conditions, demonstrating the efficiency of the presented approach.

In order to illustrate the advantages of the SDP images used in this paper for the rolling bearing fault diagnosis task, we also converted the vibration samples of rolling bearings into vibration grayscale images, short-time Fourier transform (STFT) images, and wavelet images, which are commonly used in fault diagnosis research, and carried out the comparison experiments. In the experiment, the corresponding types of image samples were firstly generated using DDPM, and then a mixed dataset was constructed by utilizing various types of real images and generated images, and the number of all samples in the dataset was set in the same way as that of the SDP images in Table 3. Finally, we performed fault diagnosis using different kinds of images as inputs and using the CNN diagnostic model proposed above, and the diagnostic results are shown in Table 5. In Table 5, the grayscale image directly converts the amplitude of the vibration signal into grayscale values obtained, the STFT image uses the Hamming window as the window function, and the wavelet image uses the Morlet wavelet as the wavelet basis function.

From the results, it can be observed that the CNN diagnostic model has the highest accuracy when using SDP images as input. This is followed by the wavelet image, STFT image, and grayscale image, respectively. This is due to the grayscale image only being obtained based on the amplitude of the vibration signal, and some fault features may be lost during the conversion process. The SFTF image allows for the analysis of the signal through a fixed-size time window, which has a limited time-frequency resolution. Wavelet images, as a result of the wavelet transform, may have boundary effects and frequency aliasing. Moreover, the wavelet transform needs to set the basis function in advance, which cannot meet the adaptive requirements of the data and has limitations in dealing with nonlinear unstable signals. Compared with the above images, an SDP image only converts the vibration signal into an SDP image in polar coordinates, as SDP images are able to contain more information concerning fault characteristics and are more suitable for carrying out rolling bearing fault diagnosis tasks.

In order to further illustrate the advantages of deep learning methods in fault diagnosis, as well as to further validate the robustness and effectiveness of the proposed methods, we conducted experimental comparisons by utilizing some widely used deep learning-based fault diagnosis models in the field of fault diagnosis. The datasets used in the experiments were all mixed datasets with sample balance reached after data supplementation using DDPM-generated images. In order to eliminate the effect of chance errors, each method was tested 10 times, and diagnostic performances were evaluated using the average accuracy of the 10 test results, which are shown in Table 6.

In the experiments, the architecture and parameter settings of the CNN diagnostic model remained unchanged. CBAM-CNN was added to the CNN model with the CBAM attention mechanism. CBAM incorporated both channel attention and spatial attention, which allowed the model to focus on regions where fault characteristics were more pronounced. The 1D-CNN model consisted of five convolutional layers, two fully connected layers, and a SoftMax classifier. The input for the 1D-CNN was a 1 × 2048 vibrating sample; the size of the convolutional kernel was 1 × 32; the number of convolutional kernels was 16, 32, 32, 64, and 128, respectively; the activation function was ReLU; and the number of nodes in the fully connected layer was 1024 and 256. The diagnostic model based on the deep belief network (DBN) consisted of one input layer, one output layer, and four hidden layers. The number of nodes in the hidden layers was 512, 256, 128, 64, and 32, and the hidden layer activation function was Sigmoid. The diagnostic model based on the stacked autoencoder (SAE) consisted of one input layer, one output layer, and four hidden layers. The number of nodes in the hidden layers was 512, 256, 128, 64, and 32, and the hidden layer activation function was ReLU.

From the experimental results, it can be seen that the CNN diagnostic method used in this paper outperforms other deep learning diagnostic methods in terms of accuracy, reaching 99.45%. This indicates that the method proposed in this paper has significant advantages over other deep learning diagnostic methods. When using the same vibration image as input, the diagnostic accuracy of CNN is significantly higher than that of DBN and SAE. This is because CNN is better able to extract the local features and spatial information in the image, which is more suitable for processing image data. Also, the diagnostic accuracy of CBAM-CNN and 1D-CNN is higher than that of DBN and SAE, which indicates that the CNN model is more capable of fault feature extraction. In addition, because the 1D vibration signals are weaker than vibration images in characterizing fault features, the diagnostic accuracy of 1D-CNN with 1D vibration signals as input is 1.72% lower than that of the method proposed in this paper. Moreover, although CBAM-CNN adds a CBAM attention mechanism to the foundation of CNN, the diagnostic accuracy of the CBAM-CNN is not significantly different from that of CNN in the proposed method in this paper or is even slightly decreased. This indicates that the sensory field of the CNN model was sufficient for fitting the target features to the data before adding the attention mechanism, so the diagnostic performance of the CNN did not change significantly after the addition of the attention mechanism. In addition, adding an attention mechanism increases the model parameters, which may increase the likelihood of overfitting in the CNN model.

It is worth noting that the diagnostic results reveal that the accuracy of all deep learning fault diagnosis methods is higher than 95%. This is because, compared with traditional shallow diagnostic models such as BPNN and SVM, deep learning models do not need to extract fault features manually, thus reducing the uncertainty of manual feature extraction. It also indicates that deep learning models have stronger feature extraction and processing capabilities, which makes them more suitable for fault diagnosis research.

Moreover, we also compared the standard deviations of the diagnostic accuracy of the different methods. The standard deviation of the accuracy rate can reflect variations in the diagnostic model’s performance in different experiments or different datasets and can indicate the robustness of the diagnostic model to some extent. From the experimental results, it can be found that the CNN diagnostic method proposed in this paper has the smallest standard deviation of accuracy, which indicates that the model has the most stable performance in multiple fault diagnostic experiments and has a strong ability to adapt to different image samples, which also indicates that the method has strong robustness.

Finally, we also compared the training time of different deep learning fault diagnosis methods. From the results, we can see that the training time of the CNN diagnostic model proposed in this paper is relatively long, but it is still within the acceptable range. The differences between the training times of CBAM-CNN, DBN, and SAE compared with CNN may be caused by the number of parameters in the model, with models with fewer parameters taking relatively less time to train. Since CNN is more suitable for processing image data, the training time of CNN will be slightly shorter than 1D-CNN. However, the training time of the fault diagnosis model is affected by many factors, such as the optimization algorithm, data size and quantity, and hardware equipment. Therefore, the training time and diagnostic performance of diagnostic models need to be evaluated in combination with specific diagnostic tasks and experimental environments.

### 4.2. Experimental Verification Based on Primary Mine Fan Rolling Bearing Data under Actual Working Conditions

When applying this method in actual working conditions, it is first necessary to carry out the long-time condition monitoring and operation status acquisition of the target equipment. The acquired normal data with a small volume of fault data were converted into SDP images. The fault images were then used to train the DDPM and enable it to generate images with similar fault characteristics as the real fault images. Next, the faulty images were supplemented using the generated images so that the number of faulty images was the same as the normal images. Finally, the CNN fault diagnosis model was trained using the balanced dataset so that it could extract the fault features and implement fault diagnosis.

In order to investigate the efficiency and applicability of the developed approach, its experimental verification was performed using the rolling bearing RB fault data of the primary mine fan under actual working conditions.

#### 4.2.1. Evaluation and Analysis of Generated Images

**Figure 15:**
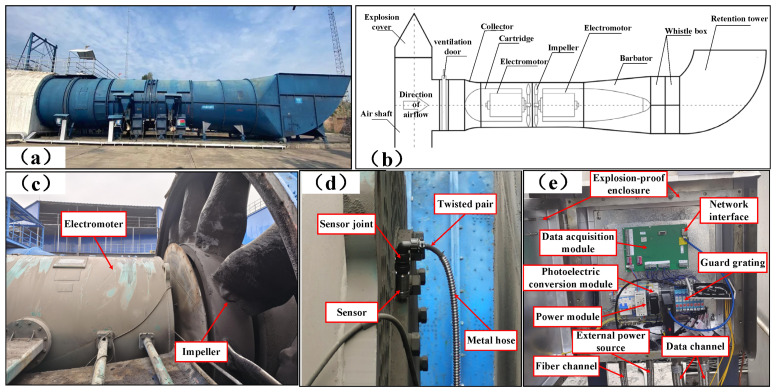
Primary mine fan and data acquisition system: (**a**) physical diagram of the primary mine fan; (**b**) schematic diagram of the main fan structure; (**c**) internal structure of the main fan; (**d**) data acquisition sensor; (**e**) data monitoring and acquisition system.

The primary mine fan rolling bearing data from an FBC(D)Z type primary mine fan in a coal mine mainly comprised a flow collector, a motor, an impeller, a diffuser, a muffler, a diffuser tower, and other structures, as presented in Figure 15a–c. The types of rolling bearings at the fan’s driving end were NU220ECM and NU330RCM, and the type of rolling bearing at the nondriving end was QJ330N2, the structural parameters of which are shown in Table 7. The FBC(D)Z-type primary mine fan ventilation method was used for extraction; its working voltage was 10 KV, its rated negative pressure was 1324–4700, and its rated air volume was 21,920 m^3^/min.

Since the vibration signal generated using the RB of the fan during operation significantly depends on the type, degree, and position of the mechanical fault of the fan, it contains a wealth of information about the equipment’s operating status. Moreover, the vibration signal-based FD approach has the advantages of easy acquisition and broad applicability. Thus, the vibration data of the fan’s RB were acquired using a vibration pulse sensor and data acquisition system, as shown in Figure 15d,e.

**Figure 16:**
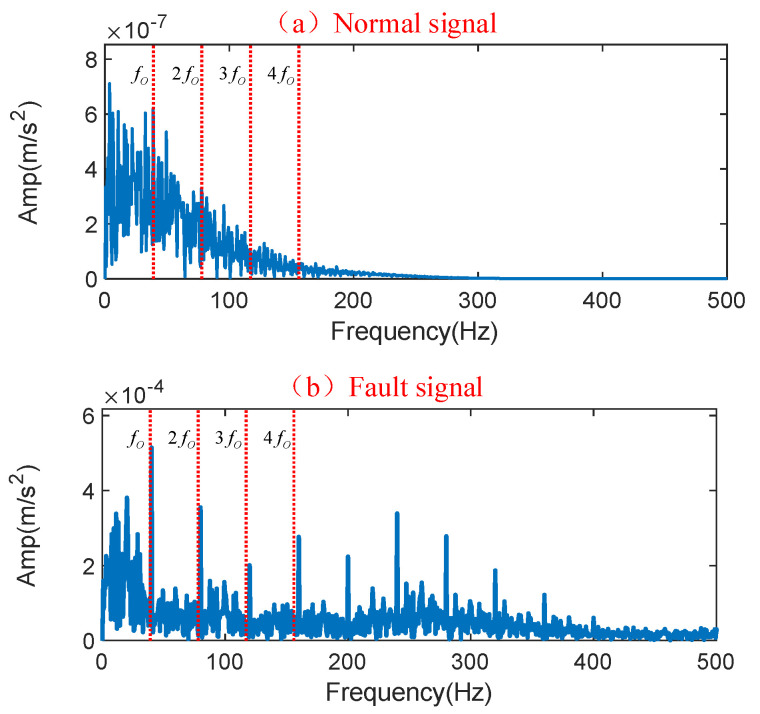
Envelope spectra of normal and fault signals.

Sensors were installed on the driving and nondriving ends of the primary mine fan motor to collect vibration signals from the rolling bearings during operation. In actual working conditions, the sensor’s sampling rate was 10 k, the resolution ratio was 0.625, the number of sampling points was 16 k, the sampling time was 1.6 s, and the signal was collected at three-hour intervals. In order to facilitate the work of repair personnel in the field, monitoring software was utilized to monitor and display the collected vibration signals. In order to evaluate the data’s truthfulness and usability, we performed signal analysis on the normal and faulty vibration signals after assessment using monitoring software. As shown in Table 8, the characteristic fault frequencies of different positions were determined by calculating the structural parameters of rolling bearings. Moreover, the two signals’ envelope spectra were obtained after signal processing, and the results are shown in Figure 16.

A frequency (40 Hz) close to the theoretical fault characteristic frequency $f_{o}$ (39.2 Hz) of the outer ring can be found in the envelope of the fault signal, and its multiplicative component is also clearly visible. This indicates that the RB has an early fault in the outer ring. In contrast, the normal signal has no multiplicative component of the characteristic fault frequency $f_{o}$. It should be noted that the deviation between the fault characteristic frequency in the fault signal and the actual signal is due to an accuracy error in the production processing and assembly process of the bearing. In summary, the vibration signal acquired using a monitoring software program can effectively reflect the rolling bearing’s operation status in the primary mine fan and can be applied to the FD of the rolling bearing in the primary mine fan.

**Figure 17:**
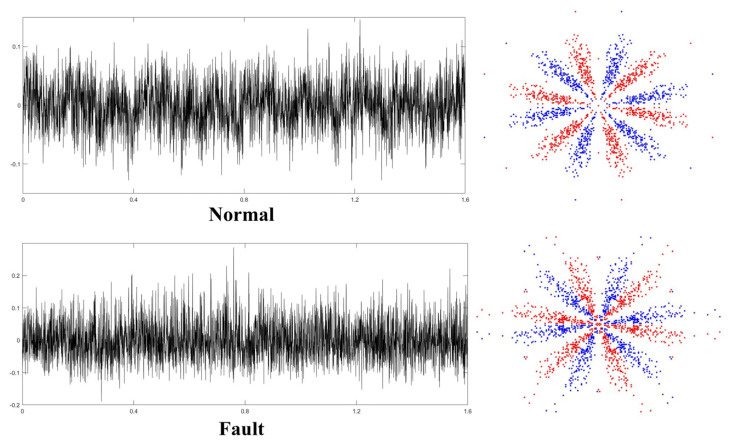
The RB vibration signals’ time-domain and SDP images in various states.

After monitoring the long-term operating conditions of the mentioned primary mine fan, the rolling bearings’ vibration signals were acquired under various operating conditions. Since the fault of the RB in the primary mine fan is derived from various factors, and the primary mine fan is different from other mechanical equipment, its ability to work properly is vital to the safety of coal production. Therefore, it must be shut down for repair once a primary mine fan fault occurs. According to the application requirements of the actual working conditions, it is only necessary to diagnose whether a fault occurs in the primary mine fan’s RB, and it is not necessary to diagnose the specific type of fault. Accordingly, we collectively defined the multiple fault operation data collected as fault status data and classified the primary mine fan rolling bearing operation data into normal and fault status. The normal and fault-state data were the majority and minority class data, respectively. First, the preprocessed bearing vibration signal was sample-segmented using the overlapping sampling method, and each sample included 512 sampling points. The SDP approach was then utilized to transform the RB vibration signal into a 2D SDP image, and the SDP image conversion parameters were similar to those outlined in the previous section. Figure 17 presents the time-domain waveforms and converted SDP images of the RB vibration signals for various working situations.

**Figure 18:**
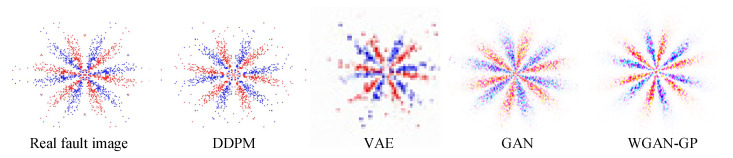
Comparison of the generated and real images.

The obtained SDP images were categorized into training and test samples, and an unbalanced sample set was established, as shown in Table 9. Fault samples as a few classes were input into DDPM, and the generated DDPM images were compared with the results of other generation methods, such as VAE, GAN, and WGAN-GP. Figure 18 presents the results. The model architecture and parameter settings of DDPM and other generation methods are similar to those seen in the previous section.

**Figure 19:**
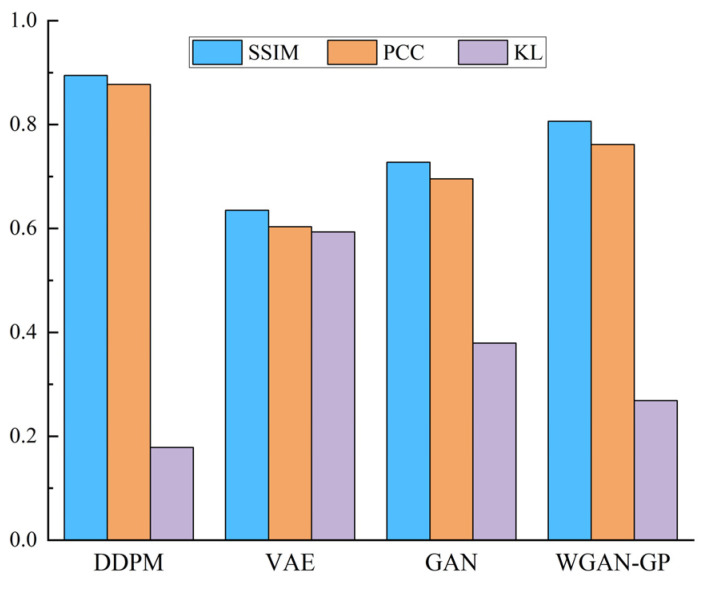
Comparing the quality of generated images based on evaluation criteria.

Figure 18 indicates that the DDPM-produced images are intuitively more similar to the real images, indicating that the quality and image similarity of the DDPM-produced images are superior to those of other generation approaches. In order to quantitatively assess the quality of the produced images, 10 generated images were selected from each of the results generated using all methods. Then, the SSIM, PCC, and KL scatters between all the generated images and the corresponding real images were counted, and the averages of the above indices were taken as the criteria for assessing the generated images’ quality, and the results are presented in Figure 19.

**Figure 20:**
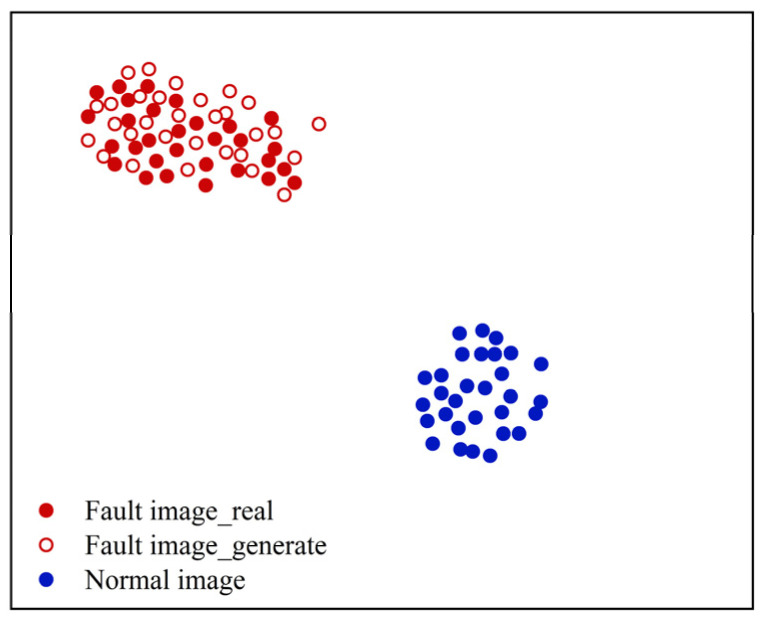
Feature visualization of the produced and actual images.

It can be observed that the presented DDPM method generates images that outperform other generation methods in all evaluation criteria. This indicates that the DDPM-generated images are of high quality and that the produced images are of similar appearance to the real images and have similar feature distributions. In order to further describe the benefits of the DDPM approach in generating images, the t-SNE approach was utilized to visualize a partial real and generated image of the primary mine fan rolling bearing fault state in a dimensionality reduction. The visualization results of the sample distribution were utilized to verify the similarity between the produced and real images. The results are presented in Figure 20.

As presented in Figure 20, the characteristics of the produced and actual images of the primary mine fan rolling bearing fault state are essentially distributed in the same area, and the overlap between them is low. This indicates that the DDPM-generated images have similar feature distributions to the real images, and DDPM is better able to fit the fault features in the real fault images. Therefore, compared with other generation methods, DDPM can generate high-quality image samples, which are able to enhance the unbalanced dataset of the primary mine fan rolling bearings.

#### 4.2.2. Application of the Sample Generation Method in Fault Diagnosis

In order to further verify that adding generated samples to the unbalanced dataset for data enhancement helps to enhance the classification precision of the RB FD model of the primary mine fan, the proposed method was validated via experiments. In the experiments, different generation methods were used to supplement the data of the imbalanced dataset shown in Table 7 to reach a balanced sample state, i.e., 200 generated images were added to the image sample of the fault state so that the number of images of the minority class (fault state) was similar to that of the majority class (normal state). The experiments were then validated using the mixed sample set shown in Table 10.

The presented CNN diagnostic model was trained using the mentioned sample set, and the CNN diagnostic model’s architecture and parameter settings were similar to those outlined in the previous section. In order to verify the efficiency of the developed approach, other FD models like SVM, RF, and BP neural networks were trained using the same dataset for comparison. The CNN FD model adopted the SDP image directly as input, while the inputs of other diagnostic models were the SDP image’s texture feature parameters. The accuracy of different datasets in different fault diagnosis models was tested using a test set. In order to alleviate the impact of chance errors, each method was tested 10 times, and their diagnostic efficiencies were evaluated through the mean precision of the 10 test results, as described in Table 11.

The experimental results indicate that the precision of the FD model in the mixed dataset is higher than that in the unbalanced dataset. This indicates that adding the generated samples to the unbalanced dataset of the primary mine fan rolling bearing for data enhancement considerably improves the precision of the FD model. It efficiently solves the problem of insufficient diagnostic accuracy induced by the unbalanced number of samples when the FD model is applied to the actual working conditions. Meanwhile, the experimental results indicate that the FD model has a higher diagnostic precision in the mixed dataset with DDPM-generated samples added than in the dataset with other generated samples added, indicating that using the DDPM method for generating a few classes of samples is a more efficient approach for data enhancement. This is because the DDPM method is able to fit the RB fault image of the primary mine fan more effectively when generating image samples, generating an image containing more effective fault features.

There is a considerable decrease in the accuracy of all diagnostic models on the primary mine fan’s rolling bearing dataset compared with the rolling bearing test set in the previous section. This is because the operating environment in the actual working conditions affects the primary mine fan, and the bearings of the primary mine fan are also subject to additional loads from the impeller and airflow. Therefore, the operating vibration signal of the RB of the primary mine fan would contain much interference noise, degrading the diagnostic precision of the FD approach. However, the CNN diagnostic model employed in the presented primary mine fan rolling bearing FD method outperforms the other commonly used diagnostic models in all datasets. Compared with the original unbalanced dataset, the presented approach improves the final FD accuracy by up to 5.9% after data enhancement. This indicates the feasibility and efficiency of both the DDPM and CNN approaches employed in the presented approach.

In order to further validate the effectiveness and robustness of the proposed method in real working conditions, we conducted comparative experiments using other commonly used deep learning fault diagnosis models. All the fault diagnosis methods had the same model architecture and parameter settings as those given in the previous section. The dataset used was also the same mixed dataset with the sample balance reached after data supplementation using DDPM-generated images. In order to eliminate the effect of chance error, each method was tested ten times and their diagnostic performances were evaluated using the average accuracy of the ten test results, which are shown in Table 12.

From the analysis of the experimental results, it can be seen that the accuracy of the CNN-based fault diagnosis method proposed in this paper is significantly higher than that of other deep learning diagnostic models, reaching 98.13%. Compared with DBN with SAE, the diagnostic accuracy of CNN is 6.1% and 3.96% higher, which indicates that the CNN-based diagnostic method is superior to the DBN with the SAE-based method in terms of diagnostic performance. Meanwhile, the diagnostic accuracy of the CNN-based method is 3.43% higher than that of the 1D-CNN-based method, and this result likewise illustrates the greater advantage of fault diagnosis research based on vibration images and the superiority of the proposed method in this paper. Moreover, the diagnostic accuracy of the CNN diagnostic model before and after the addition of CBAM does not differ much, indicating that the CNN model is sufficient for fitting the target data for the current diagnostic task, so its diagnostic performance remains basically unchanged.

Comparing the experimental results with the diagnostic results of conventional rolling bearings in the previous section, all deep learning diagnostic methods showed a decrease in accuracy for the rolling bearing data in the primary mine fan, especially the 1D-CNN-based diagnostic method. This is due to the presence of a large amount of interfering noise in the vibration signals of the rolling bearings in the primary mine fan, which masks some of the fault characteristics, thus affecting the diagnostic performance. However, the diagnostic performance of the proposed method still achieves satisfactory results, which validates and illustrates the effectiveness and robustness of the proposed method in this paper. Meanwhile, the accuracy of other methods is also higher than 90%, which illustrates the superiority of deep learning methods in fault diagnosis research.

To further validate the robustness of the proposed methods, we calculated the standard deviations of the accuracy rates of all diagnostic methods over multiple experiments. The results show that the CNN diagnostic method proposed in this paper has the smallest variation in accuracy rate over multiple experiments, and its standard deviation is also the smallest. This confirms that the proposed method is robust, and it has a strong adaptive ability in dealing with different data or in the presence of noise and still maintains a relatively stable diagnostic performance.

Moreover, it can be seen from the training time of all fault diagnostic models that the training time of diagnostic models increases when dealing with real data under actual working conditions. The reason for this phenomenon may be the presence of a large amount of noise interference in the real vibration signal, which can increase the difficulty of the model when extracting fault features. However, the training times of CNN and other deep learning fault diagnosis methods are within acceptable limits, indicating that deep learning methods are more suitable for fault diagnosis tasks under certain conditions. The analysis results reveal that the FD approach using the SDP image, DDPM, and CNN model presented in the current work can precisely detect and classify the operation status of the rolling bearings in the primary mine fan. This demonstrates the efficiency of the presented approach and shows that it can be appropriately applied to the condition monitoring and FD of the primary mine fan rolling bearings under actual working conditions.

## 5. Conclusions

The current study presents a primary mine fan rolling bearing FD approach using SDP images, the DDPM image generation method, and the CNN, which are able to accurately identify the primary mine fan rolling bearing fault under sample imbalance conditions. Using the SDP method, the 1D vibration signal was first converted into a distinctly characterized 2D SDP image. The DDPM method was then utilized to generate images with similar feature distributions to a minority class of real fault images, which were then added to the imbalanced dataset for data enhancement. Finally, taking advantage of the CNN in image processing, the CNN was adopted as a fault diagnosis model to identify and classify the working situations of RBs.

In order to assess the efficiency of the presented approach, it was validated using a conventional RB fault test bench dataset in a laboratory environment and the real operating data of the primary mine fan rolling bearings under actual working situations. In the experiments, the correlation coefficients between the produced and actual images were determined to investigate the optimal training rounds of DDPM. The quality of DDPM-produced images was then evaluated quantitatively using various evaluation metrics and compared with other popular image generation approaches. Finally, the effects of the number of generated samples on the precision of the FD model under various sample imbalance rates and the effects of data enhancement on the FD precision were compared and analyzed using different image generation methods for imbalanced datasets.

Although this approach attained relatively excellent results in the sample imbalance FD of the primary mine fan rolling bearing, in future works, further areas should be explored, such as improving the sampling speed of the image generation method, enhancing the FD method’s anti-interference capabilities regarding the severe noise in real operating situations, and the research of a lightweight diagnosis model for various applications.

## Figures and Tables

**Table 1:** Unbalanced dataset of rolling bearing fault samples.

Fault Status	Training Set	Test Set
Normal	1500	200
Inner-ring fault	1000	200
Outer-ring fault	1000	200
Rolling-element fault	1000	200

**Table 2:** Mixed dataset of rolling bearing samples at various imbalance rates.

Experiment Number	Number of Training Samples		Number of Test Samples	Average Accuracy (%)
	Real Samples	Generate Samples		
1	1000	0	800	98.45
2	500	0	800	97.5
3	100	0	800	88.5
4	1000	5000	800	94.85
5	1000	3000	800	97.6
6	1000	1500	800	98.75
7	1000	1250	800	98.95
8	1000	1000	800	99.15
9	1000	750	800	99.3
10	1000	500	800	99.45
11	1000	250	800	99.05
12	1000	100	800	98.55
13	500	5000	800	91.35
14	500	2500	800	92.5
15	500	1000	800	96.85
16	500	500	800	98.05
17	500	250	800	98.35
18	500	100	800	98.25
19	100	100	800	88.85
20	0	1000	800	97.8
21	0	500	800	95.05
22	0	100	800	86.9

**Table 3:** Mixed dataset of rolling bearing samples.

Fault Status	Training Set		Test Set
	Real Images	Generate Images	
Normal	1500	0	200
Inner-ring fault	1000	500	200
Outer-ring fault	1000	500	200
Rolling-element fault	1000	500	200

**Table 4:** Average classification precision of various FD approaches.

Diagnosis Method	Unbalanced Dataset	Mixed Dataset after the Addition of Generated Samples			
		DDPM	VAE	GAN	WGAN-GP
CNN	93.3	99.45	94.15	94.85	96.85
SVM	81.05	88.25	83.55	83.2	85.85
RF	82.5	86.4	83.95	84.15	85.05
BP	68.15	73.95	70.4	70.85	72.45

**Table 5:** Effect of different input images for diagnostic accuracy.

Input Images	SDP Images	Grayscale Images	STFT Images	Wavelet Images
Average accuracy (%)	99.45	96.63	97.75	98.63

**Table 6:** Average diagnostic accuracy of different deep learning fault diagnosis methods.

Diagnostic Methods	Average Accuracy (%)	Standard Deviation (%)	Training Time (s)
CNN	99.45	0.2784	536.72
CBAM-CNN	99.41	0.3115	584.96
1D-CNN	97.73	0.5683	677.3
DBN	95.15	0.6784	517.85
SAE	97.1	0.6442	462.57

**Table 7:** Mining main ventilation fan rolling bearing structure parameters.

Bearing Type	NU220ECM	NU330RCM	QJ330N2
Rolling elements’ count	17	14	11
Inner-ring diameter	100 mm	150 mm	150 mm
Outer-ring diameter	180 mm	320 mm	320 mm
Width	34 mm	65 mm	65 mm
Rolling elements’ diameter	22 mm	42 mm	45 mm
Contact angle	0°	0°	15°

**Table 8:** Frequency of fault characteristics.

Bearing Type	Rolling Element ($f_{r}$)	Outer Ring ($f_{o}$)	Inner Ring ($f_{i}$)
NU220ECM	27.36 Hz	62.77 Hz	85.97 Hz
NU330RCM	22.31 Hz	49.66 Hz	72.83 Hz
QJ330N2	15.34 Hz	39.2 Hz	57.03 Hz

**Table 9:** Unbalanced dataset of rolling bearings of the primary mine fan.

Operation Status	Training Set	Test Set
Normal Status	600	150
Fault status	400	150

**Table 10:** Mixed dataset of the primary mine fan rolling bearing samples.

Operation Status	Training Set		Test Set
	Real Images	Generate Images	
Normal Status	600	0	150
Fault status	400	200	150

**Table 11:** Mean classification precision of various FD methods.

Diagnosis Method	Unbalanced Dataset	Mixed Dataset after Adding Generated Samples			
		DDPM	VAE	GAN	WGAN-GP
CNN	92.23	98.13	92.83	94.17	96.07
SVM	78.57	85.73	80.13	81.83	83.43
RF	77.17	84.33	79.63	82.17	82.97
BP	54.13	63.6	57.67	58.67	61.47

**Table 12:** Diagnostic accuracy of different methods for fan-bearing data.

Diagnostic Methods	Average Accuracy (%)	Standard Deviation (%)	Training Time (s)
CNN	98.13	0.31	594.18
CBAM-CNN	97.93	0.3667	635.74
1D-CNN	94.7	0.6046	694.87
DBN	92.03	0.8492	583.15
SAE	94.17	1.0878	504.67

## Data Availability

Data are available upon reasonable request, subject to restrictions (e.g., privacy or ethical restrictions).
